# Segregation of the human medial prefrontal cortex in social cognition

**DOI:** 10.3389/fnhum.2013.00232

**Published:** 2013-05-29

**Authors:** Danilo Bzdok, Robert Langner, Leonhard Schilbach, Denis A. Engemann, Angela R. Laird, Peter T. Fox, Simon B. Eickhoff

**Affiliations:** ^1^Institute of Neuroscience and Medicine (INM-1), Research Center JülichJülich, Germany; ^2^Institute of Clinical Neuroscience and Medical Psychology, Heinrich Heine UniversityDüsseldorf, Germany; ^3^Max-Planck-Institute for Neurological ResearchCologne, Germany; ^4^Department of Psychiatry, University of CologneCologne, Germany; ^5^Institute of Neuroscience and Medicine (INM-3), Research Center JülichJülich, Germany; ^6^Department of Physics, Florida International UniversityMiami, FL, USA; ^7^Research Imaging Institute, University of Texas Health Science CenterSan Antonio, TX, USA; ^8^South Texas Veterans Administration Medical CenterSan Antonio, Texas

**Keywords:** social cognition, medial prefrontal cortex, meta-analytic connectivity modeling, resting state connectivity, functional decoding, data-mining

## Abstract

While the human medial prefrontal cortex (mPFC) is widely believed to be a key node of neural networks relevant for socio-emotional processing, its functional subspecialization is still poorly understood. We thus revisited the often assumed differentiation of the mPFC in social cognition along its ventral-dorsal axis. Our neuroinformatic analysis was based on a neuroimaging meta-analysis of perspective-taking that yielded two separate clusters in the ventral and dorsal mPFC, respectively. We determined each seed region's brain-wide interaction pattern by two complementary measures of functional connectivity: co-activation across a wide range of neuroimaging studies archived in the BrainMap database and correlated signal fluctuations during unconstrained (“resting”) cognition. Furthermore, we characterized the functions associated with these two regions using the BrainMap database. Across methods, the ventral mPFC was more strongly connected with the nucleus accumbens, hippocampus, posterior cingulate cortex, and retrosplenial cortex, while the dorsal mPFC was more strongly connected with the inferior frontal gyrus, temporo-parietal junction, and middle temporal gyrus. Further, the ventral mPFC was selectively associated with reward related tasks, while the dorsal mPFC was selectively associated with perspective-taking and episodic memory retrieval. The ventral mPFC is therefore predominantly involved in bottom-up-driven, approach/avoidance-modulating, and evaluation-related processing, whereas the dorsal mPFC is predominantly involved in top–down-driven, probabilistic-scene-informed, and metacognition-related processing in social cognition.

## Introduction

Functional specialization in the human prefrontal cortex has been investigated since the middle of the nineteenth century primarily by lesion reports (Harlow, 1848, 1868; Broca, 1865). However, hard evidence derivable from functional double dissociations by prefrontal brain lesions is rare in humans (cf. Gaffan, 2002; Wilson et al., 2010). Nevertheless, the parts of the prefrontal cortex are known to be involved in many high-level cognitive functions, including executive control, action selection, multi-tasking, social cognition, or general intelligence. These disparate roles have been parsimoniously explained by different concepts, including the conjoint consideration of internal subtasks, branching and reallocation of attention, or balancing between self-generated and environmental information. Yet, there may be no common denominator for all functional involvements of the PFC (Wood and Grafman, 2003; Ramnani and Owen, 2004; Amodio and Frith, 2006; Burgess et al., 2006; Koechlin and Hyafil, 2007; Forbes and Grafman, 2010; O'Reilly, 2010).

In contrast, activity changes in medial aspects of the prefrontal cortex (mPFC) were frequently related to social cognition, defined as information processing related to human individuals as opposed to the physical world. Examples of such functional involvements include processing affective information (Phan et al., 2002), forming social judgments (Freeman et al., 2010; Bzdok et al., 2012b), attributing beliefs (den Ouden et al., 2005), retrieving social semantic knowledge (Contreras et al., 2012), and encountering unstable social hierarchies (Zink et al., 2008). In fact, Mitchell (2009) noted that the core domains of social psychology converge exclusively in the mPFC, rendering this scientific field naturally coherent rather than an arbitrary outcome of historical evolution. In social neuroscience, most propositions for functional specialization of the mPFC relied on the distinction between a ventral and a dorsal functional compartment. More specifically, ventral versus dorsal mPFC regions (vmPFC/dmPFC) have been variously proposed to be functionally dissociable according to emotional versus cognitive, automatic versus controlled, implicit versus explicit, outcome-oriented versus goal-oriented, or self-relevant versus other-relevant social cognition (Amodio and Frith, 2006; Mitchell et al., 2006; Shamay-Tsoory et al., 2006; Lieberman, 2007; Olsson and Ochsner, 2008; Van Overwalle, 2009; Forbes and Grafman, 2010). The diversity of proposed functional dissociations between the vmPFC and dmPFC illustrates the current lack of consensus.

In the current study, we therefore quantitatively examined the functional organization of the mPFC along its ventrodorsal axis. First, the analysis was based on two seed regions in the vmPFC and dmPFC, respectively. These regions corresponded to locations showing significant convergence of perspective-taking tasks in a recent coordinate-based meta-analysis (Bzdok et al., 2012c). As perspective-taking is probably a uniquely human capacity (Premack and Woodruff, 1978; Tomasello et al., 2003), these two clusters of underlying convergent activity are an excellent proxy for the different functional compartments of the mPFC in human social cognition in general. Second, we delineated brain-wide connectivity of each seed according to two complementary measures of functional connectivity, task-dependent meta-analytic connectivity modeling (MACM, Eickhoff et al., 2011) and task-independent resting state correlations (RS, Biswal et al., 1995). MACM analysis is based on co-activation patterns across a large number of databased neuroimaging experiments (i.e., brain activity under task constraints). RS analysis, in turn, is based on correlations of slow (<0.1 Hz) fluctuations of fMRI signals during rest (i.e., unconstrained brain activity in the absence of an externally purported task). Third, we determined a functional profile for each seed using BrainMap meta-data (Laird et al., 2011) by complementary forward and reverse functional decoding. This approach allowed for a cross-validated connectional and functional segregation of the ventral and dorsal mPFC segregation as involved in social cognition.

## Methods

### Definition of the seed regions

We conducted connectivity analyses and functional profiling of two seed regions in the mPFC that were derived from a recent coordinate-based meta-analysis (Bzdok et al., 2012c) using the activation-likelihood estimation (ALE) algorithm (Eickhoff et al., 2009, 2012; Eickhoff and Bzdok, 2012). This meta-analysis quantitatively summarized all neuroimaging experiments related to perspective-taking published until 2010, in all, 68 experiments reporting 724 activation foci (Bzdok et al., 2012c). It included neuroimaging experiments [fMRI and positron emission tomography (PET)] in which participants were required to adopt an intentional stance towards others, that is, predict their thoughts, intentions, and future actions. It excluded neuroimaging experiments using non-whole-brain analyses, pharmacological manipulations, or psychiatrically/neurologically diagnosed individuals. More specifically, the two chosen seed regions represent regions of converging brain activity revealed by the (cluster-level corrected) quantitative meta-analysis of neuroimaging results from various paradigms that prompt perspective-taking. Please note that the meta-analyses on empathy and morality, also reported in that meta-analytic study, did not contribute to our seeds. The previously published meta-analysis on perspective-taking thus yielded two continuous, non-overlapping clusters of convergent brain activity that served as neuroanatomical constraints for the differential localization of higher social processes in the mPFC. Put differently, those seeds reflect, first, two topographically constrained brains areas closely related to social processes and, second, the widely assumed functional segregation in this area in the neuroimaging literature on social cognition (e.g., Mitchell et al., 2006; Shamay-Tsoory et al., 2006; Van Overwalle, 2009). Each cluster's whole-brain connectivity pattern was subsequently delineated by task-dependent meta-analytic connectivity modeling and task-independent resting-state analyses. As the employed meta-analytic seeds naturally have asymmetrical shapes we repeated all analyses after fusion of the original seeds with the sagitally mirrored seeds, which yielded virtually identical results.

### Task-dependent functional connectivity: MACM

The delineation of whole-brain co-activation maps for each seed was performed based on the BrainMap database (www.brainmap.org; Fox and Lancaster, 2002; Laird et al., 2011). We constrained our analysis to “normal” fMRI and PET experiments (i.e., no pharmacological interventions, no group comparisons) in healthy participants, which report whole-brain results as coordinates in a standard stereotaxic space. These inclusion criteria yielded ~6500 eligible experiments at the time of analysis. Note that we considered all eligible BrainMap experiments because any pre-selection based on taxonomic categories would have constituted a strong *a priori* hypothesis about how different tasks etc. involve different brain networks. Yet, it remains elusive how well psychological constructs, such as emotion and cognition, map on regional brain responses (Mesulam, 1998; Poldrack, 2006; Laird et al., 2009a). To reliably determine the co-activation patterns of a given seed, we identified the set of experiments in BrainMap that reported at least one activation focus within that seed. The brain-wide co-activation pattern for each seed was then computed by ALE meta-analysis over (all foci reported in) the experiments that were associated with that particular seed (Turkeltaub et al., 2002; Eickhoff et al., 2009; Laird et al., 2009a). The key idea behind ALE is to treat the foci reported in the associated experiments not as single points, but as centers for 3D Gaussian probability distributions that reflect the spatial uncertainty associated with neuroimaging results. Using the latest ALE implementation (Eickhoff et al., 2009, 2012; Turkeltaub et al., 2012), the spatial extent of those Gaussian probability distributions was based on empirical estimates of between-subject and between-template variance of neuroimaging foci (Eickhoff et al., 2009). For each experiment, the probability distributions of all reported foci were then combined into a modeled activation (MA) map by the recently introduced “non-additive” approach that prevents local summation effects (Turkeltaub et al., 2012). The voxel-wise union across the MA maps of all experiments associated with a particular seed voxel then yielded an ALE score for each voxel of the brain that describes the co-activation probability of that particular location with the current seed voxel.

To establish which regions were significantly co-activated with a particular seed, ALE scores for the MACM analysis of this seed were compared to a null-distribution that reflects a random spatial association between experiments, but regards the within-experiment distribution of foci as fixed (Eickhoff et al., 2009). This random-effects inference assesses above-chance convergence between experiments. The observed ALE scores from the actual meta-analysis of experiments activating within a particular seed were then tested against the ALE scores obtained under this null-distribution yielding a p-value based on the proportion of equal or higher random values (Eickhoff et al., 2012). The resulting p-values were then thresholded at *p* < 0.05 with cluster-level family-wise error correction for multiple comparisons (cluster-forming threshold at voxel-level: *p* < 0.001).

Differences in co-activation patterns between the seeds were assessed by first performing MACM separately on the experiments associated with either seed and computing the voxel-wise difference between the ensuing ALE maps (Eickhoff et al., 2011). All experiments contributing to either analysis were then pooled and randomly divided into two groups of the same size as the two original sets of experiments. That is, if 100 experiments in BrainMap featured activation in seed A and 75 featured activation in seed B, the resulting pool of (175) experiments would be randomly divided into a group of 100 and a group of 75 experiments. ALE-scores for these two randomly assembled groups were calculated and the difference between these ALE-scores was recorded for each voxel in the brain. Repeating this process 10,000 times yielded an empirical null-distribution for the differences in ALE-scores between the MACM analyses of the two seeds. The observed difference in ALE scores was then tested against this null-distribution yielding a p-value for the difference at each voxel based on the proportion of equal or higher random differences. The resulting non-parametric p-values were thresholded at *p* > 0.95 and inclusively masked by the respective main effects, i.e., the already thresholded effects from the MACM analysis of the particular seed, to focus inference on regions reliably co-activating with that seed.

### Task-independent functional connectivity: RS correlations

Next, seed-wise whole-brain connectivity was assessed using resting-state correlations as an independent modality of functional connectivity. This analysis was based on RS fMRI data from 139 healthy volunteers (56 female, mean age 42.3 years) without any record of neurological or psychiatric disorders. This dataset was obtained through the 1000 Functional Connectomes Project as part of the NKI/Rockland sample (http://fcon_1000.projects.nitrc.org/indi/pro/nki.html). Participants were instructed to keep their eyes closed and just let their mind wander without thinking of anything in particular but not to fall asleep. For each participant, 260 RS echo-planar imaging (EPI) volumes were acquired on a Siemens TimTrio 3T scanner using blood-oxygen-level-dependent (BOLD) contrast [gradient-echo EPI pulse sequence, TR = 2.5 s, TE = 30 ms, flip angle = 80°, in-plane resolution = 3.0 × 3.0 mm^2^, 38 axial slices (3.0 mm thickness) covering the entire brain]. The first four scans served as dummy images allowing for magnetic field saturation and were discarded prior to further processing using SPM8 (www.fil.ion.ucl.ac.uk/spm). The EPI images were first corrected for head movement by affine registration using a two-pass procedure. The mean EPI image for each participant was then spatially normalized to the MNI single-subject template (Holmes et al., 1998) using the ‘unified segmentation’ approach (Ashburner and Friston, 2005) and the ensuing deformation was applied to the individual EPI volumes. Finally, images were smoothed by a 5-mm FWHM Gaussian kernel to improve signal-to-noise ratio and compensate for residual anatomical variations.

The time-series data of each individual seed voxel were processed as follows (zu Eulenburg et al., 2012; Satterthwaite et al., 2013): In order to reduce spurious correlations, variance that could be explained by the following nuisance variables was removed: (1) The six motion parameters derived from the image realignment, (2) the first derivative of the realignment parameters, and (3) mean gray-matter, white-matter, and cerebrospinal fluid signal per time-point as obtained by averaging across voxels attributed to the respective tissue class in the SPM eight segmentation. All of these nuisance variables entered the model as first- and second-order terms (Jakobs et al., 2012; Reetz et al., 2012; Satterthwaite et al., 2013). Data were then band-pass filtered preserving frequencies between 0.01 and 0.08 Hz since meaningful resting-state correlations will predominantly be found in these frequencies given that the BOLD response acts as a low-pass filter (Biswal et al., 1995; Fox and Raichle, 2007).

According to this procedure, time courses were extracted for all voxels of a given seed of the individual participant and the time course of the entire seed was then expressed as the first eigenvariate of its voxels' time courses. Pearson correlation coefficients between the time series of the seeds and all other gray-matter voxels in the brain were computed to quantify RS connectivity. These voxel-wise correlation coefficients were then transformed into Fisher's Z-scores and tested for consistent deviation from zero across participants in a random-effects analysis. In particular, the Fisher's Z transformed whole-brain connectivity maps of all seeds were included in an ANOVA accounting for non-sphericity in the data originating from the fact that the different seeds represented correlated measures within each subject with unequal variance between seeds and subjects. Appropriate linear contrasts were then applied to test for regions significantly connected to the seed in the ventral and dorsal mPFC, respectively. The results of this random-effects difference analysis were cluster-level thresholded at *p* < 0.05 (cluster-forming threshold at voxel-level: *p* < 0.001), analogous to the MACM-based difference analysis.

### Conjunction and difference analyses across both connectivity modalities

To identify brain areas showing convergent task-dependent and task-independent functional connectivity with an individual seed, we performed a conjunction analysis across the MACM- and RS-derived (cluster-level corrected) connectivity maps using the strict minimum statistics (Nichols et al., 2005; Jakobs et al., 2012). Thus, surviving voxels were functionally associated with a given seed in both task-constrained (“focused”) and task-unconstrained (“resting”) brain states.

The main focus was, however, on connectivity differences between the vmPFC and dmPFC seeds. To this aim, we identified regions with significantly stronger coupling with either seed across task-dependent and task-independent functional connectivity. That is, we computed the conjunction (across both connectivity modalities) of the contrasts (between seeds) to determine regions that were more strongly connected to the ventral or dorsal seed across two disparate brain states (Cieslik et al., 2012; Reetz et al., 2012; Rottschy et al., 2012).

### Functional profiling of the seeds

The functional characterization of the two mPFC seeds was based on the BrainMap meta-data that describe each neuroimaging experiment included in the database. Behavioral domains code the mental processes isolated by the statistical contrasts (Fox et al., 2005) and comprise the main categories cognition, action, perception, emotion, and interoception, as well as their related sub-categories. Paradigm classes categorize the specific task employed (Turner and Laird, 2012; for the complete BrainMap taxonomy, see http://brainmap.org/scribe/).

*Forward inference* on the functional characterization then tests the probability of observing activity in a brain region given knowledge of the psychological process, whereas *reverse inference* tests the probability of a psychological process being present given knowledge of activation in a particular brain region (Poldrack, 2006; Yarkoni et al., 2011). In the forward inference approach, a cluster's functional profile was determined by identifying taxonomic labels for which the probability of finding activation in the respective cluster was significantly higher than the overall chance (across the entire database) of finding activation in that particular cluster. Significance was established using a binomial test (*p* < 0.001; Eickhoff et al., 2011). In the reverse inference approach, a cluster's functional profile was determined by identifying the most likely behavioral domains and paradigm classes given activation in a particular cluster. Significance was then assessed by means of a chi-square test (*p* < 0.001). Base rates for activations in the respective clusters as well as base rates for tasks were taken into account using the Bayesian formulation for deriving P(Task|Activation) based on P(Activation|Task) as well as P(Task) and P(Activation). In sum, forward inference assesses the probability of activation given a psychological term, while reverse inference assesses the probability of a psychological term given activation (Cieslik et al., 2012; Reetz et al., 2012; Rottschy et al., 2012; Kellermann et al., 2013).

The contrast analyses between the two seeds' functional profiles, in turn, were constrained to those experiments in BrainMap activating either seed. That is, the task associations of experiments in this composite pool were quantified in comparison between the respective seeds and thresholded at *p* < 0.05 (false-discovery-rate corrected for multiple comparisons). Forward inference here compared the activation probabilities between the two seeds given a particular psychological term, while reverse inference compared the probabilities of a particular psychological term being present given activation in one or the other seed. Please note that the contrast analysis results were masked with the respective individual functional decoding results of either seed. Put differently, a psychological term can only be significantly more associated with a seeds, if it was also determined significant in the main effect of functional decoding of that seed. Finally, conjunction analyses across the two seeds' functional profiles tested for significant associations of each particular psychological term with both seeds.

Notably, this approach aims at relating defined psychological tasks to the examined brain regions instead of claiming “a unique role” of a brain region for any psychological task (Mesulam, 1998; Poldrack, 2006; Yarkoni et al., 2011). Put differently, an association of task X to brain region Y obtained in these analyses does not necessarily imply that neural activity in region Y *is limited to* task X.

## Results

### Functional connectivity: individual analyses of seeds

**Figure 1:**
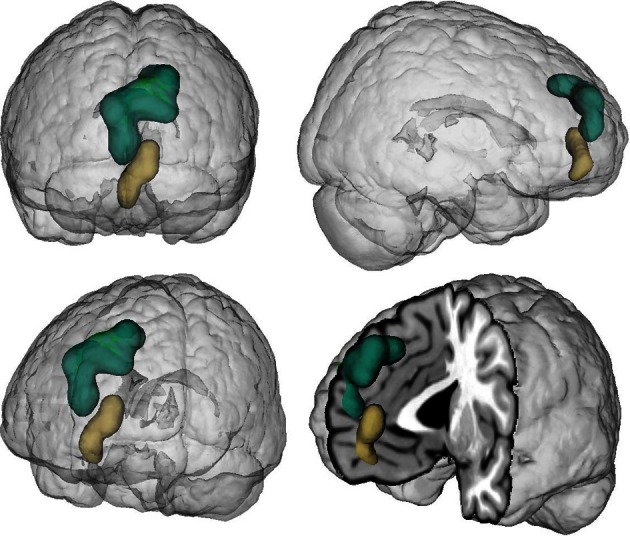
**Location of the seed regions.** Seeds were drawn from an earlier coordinate-based neuroimaging meta-analysis on perspective-taking, which yielded two clusters of convergent brain activity in the ventral (beige) and dorsal (green) medial prefrontal cortex (Bzdok et al., 2012c). The centers of mass of the vmPFC and dmPFC seed are −4/52/−2 and −6/56/30, respectively. These two seeds represent a functional-structural segregation in the medial prefrontal cortex related to higher social-cognitive processing and provided the basis for the present quantitative analyses. The seeds were rendered into a T1-weighted MNI single subject template using mango (multi-image analysis GUI; http://ric.uthscsa.edu/mango/).

**Figure 2:**
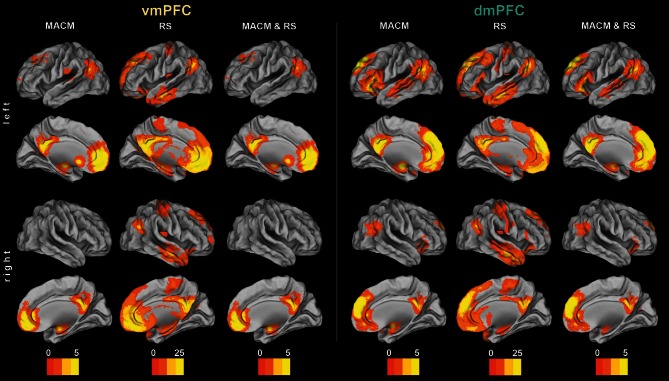
**Functional connectivity of the vmPFC and dmPFC seeds.** Connectivity patterns of each seed as individually determined using meta-analytic connectivity modeling (MACM) and resting-state (RS) analyses. The color bars on the bottom represent *Z*-values. All results survived a cluster-corrected threshold of *p* < 0.05. Please refer to Tables 1, 2 for peak coordinates. All images were rendered using Caret (computer assisted reconstruction and editing toolkit; http://brainvis.wustl.edu/wiki/index.php/Caret: About). Cortical sheet inflation enhances visual intuitiveness and alleviates activation burying in sulci.

We first determined each seed's (Figure 1) functional connectivity separately by means of both task-dependent MACM and task-independent RS analyses (Figure 2 and Tables 1, 2). MACM analysis of the vmPFC seed yielded the bilateral vmPFC and dmPFC extending into the anterior cingulate cortex (ACC), amygdala/hippocampus (AM/HC), posterior cingulate cortex/retrosplenial cortex (PCC/RSC), as well as the left nucleus accumbens (NAc), temporo-parietal junction (TPJ), superior frontal gyrus, and posterior operculum (pOP). RS analysis of the vmPFC seed yielded the bilateral vmPFC and dmPFC extending into the ACC, AM, HC, NAc, posterior mid-cingulate cortex (pMCC), RSC/PCC, precuneus (Prec), TPJ, middle temporal gyrus (MTG), temporal pole (TP), precentral gyrus (PreG), pOP, and cerebellum (Cer, not depicted) as well as the right postcentral gyrus (PoG). MACM analysis of the dmPFC seed, in turn, yielded the bilateral vmPFC and dmPFC extending into the ACC, AM/HC, inferior frontal gyrus (IFG), PCC/RSC, TPJ, and TP, as well as the left anterior insula (AI) and MTG. RS analysis of the dmPFC seed yielded the bilateral vmPFC and dmPFC extending into the ACC, AM, HC, IFG, pMCC, PCC/RSC, Prec, TPJ, MTG, TP, PreG, PoG, pOP, and Cer (not depicted).

**Table 1:** **Functional connectivity of the vmPFC seed**.

**Macroanatomical location**	***x***	***y***	***z***	***Z***
**MACM(vmPFC)**				
Ventromedial prefrontal cortex	0	52	−8	8.7
Dorsomedial prefrontal cortex	−12	48	24	3.7
Right amygdala/hippocampus	24	−6	−20	6.8
Left amygdala/hippocampus	−22	−14	−18	7.4
Left nucleus accumbens	−8	14	−6	5.8
Posterior cingulate cortex	0	−42	36	5.6
Retrosplenial cortex	−2	−52	30	6.7
Left temporo−parietal junction	−48	−66	28	5.9
Left superior frontal gyrus	−18	38	46	4.5
Left posterior operculum	−60	−28	18	6.7
**RS(vmPFC)**				
Ventromedial prefrontal cortex	−2	50	−10	31.9
Dorsomedial prefrontal cortex	0	51	17	17.6
Right amygdala	19	−1	−20	6.7
Left amygdale	−16	−1	−21	6.6
Right hippocampus	24	−20	−20	15.0
Left hippocampus	−30	−30	−12	12.9
Right nucleus accumbens	7	13	−11	12.1
Left nucleus accumbens	−4	12	−11	12.7
Posterior mid−cingulate cortex	2	−17	39	15.0
Posterior cingulate cortex	−2	−44	30	21.9
Retrosplenial cortex	6	−50	22	22.5
Precuneus	3	−70	63	15.6
Right temporo−parietal junction	46	−68	28	14.0
Left temporo−parietal junction	−48	−68	38	14.6
Right middle temporal gyrus	62	−6	−24	14.8
Left middle temporal gyrus	−66	−14	−24	14.9
Right temporal pole	42	20	−34	9.0
Left temporal pole	−44	22	−40	8.4
Right precentral gyrus	34	−26	48	8.6
Left precentral gyrus	−36	−24	54	6.9
Right postcentral gyrus	38	−30	54	7.6
Right posterior operculum	38	−22	18	6.6
Left posterior operculum	−44	−18	18	5.4
Right cerebellum	52	−66	−42	9.5
Right cerebellum	6	−54	−46	11.4
Left cerebellum	−36	−78	−38	9.5
Left cerebellum	−6	−56	−46	10.1
**MACM and RS(vmPFC)**				
Ventromedial prefrontal cortex	0	52	−8	8.7
Dorsomedial prefrontal cortex	−18	38	46	4.5
Right amygdala/hippocampus	24	−8	−20	6.6
Left amygdala/hippocampus	−22	−14	−18	7.4
Left nucleus accumbens	−8	14	−6	5.8
Posterior cingulate cortex	0	−42	36	5.6
Retrosplenial cortex	−2	−52	30	6.7
Left temporo−parietal junction	−48	−66	28	5.9
Left superior frontal gyrus	−18	38	46	4.5

Table shows coordinates derived from respective cluster peaks (x, y, z) and Z-scores (Z).

**Table 2:** **Functional connectivity of the dmPFC seed**.

**Macroanatomical location**	***x***	***y***	***z***	***Z***
**MACM(dmPFC)**				
Ventromedial prefrontal cortex	−4	48	−12	7.5
Dorsomedial prefrontal cortex	2	56	24	8.7
Right amygdala/hippocampus	20	−4	−16	5.5
Left amygdala/hippocampus	−22	−6	−18	6.9
Right inferior frontal gyrus	42	26	−7	4.2
Left inferior frontal gyrus	−48	26	−6	8
Left anterior insula	−32	24	−2	4.2
Posterior cingulate cortex	−4	−48	32	8.4
Retrosplenial cortex	−6	−56	8	5.1
Right temporo-parietal junction	54	−70	20	6.4
Left temporo-parietal junction	−52	−68	16	7.0
Left middle temporal gyrus	−60	−36	2	5.5
Right temporal pole	40	16	−20	4.4
Left temporal pole	−36	20	−24	4.5
**RS(dmPFC)**				
Ventromedial prefrontal cortex	3	43	−23	17.7
Dorsomedial prefrontal cortex	−8	56	28	26.7
Right amygdala	18	−6	−20	5.0
Left amygdale	−20	−4	−20	7.8
Right hippocampus	26	−18	−22	7.7
Left hippocampus	−26	−20	−18	10.0
Right inferior frontal gyrus	38	30	−18	10.1
Left inferior frontal gyrus	−56	29	3	9.3
Posterior mid-cingulate cortex	−2	−16	38	14.8
Posterior cingulate cortex	−4	−46	34	21.5
Retrosplenial cortex	6	−50	24	17.8
Precuneus	−1	−64	33	15
Right temporo-parietal junction	54	−66	26	14.3
Left temporo-parietal junction	−52	−60	26	18.3
Right middle temporal gyrus	62	−6	−26	15.7
Left middle temporal gyrus	−66	−8	−22	16.7
Right temporal pole	46	14	−36	13.1
Left temporal pole	−52	10	−38	13.9
Right precentral gyrus	32	−28	50	9.7
Left precentral gyrus	−30	−28	58	8
Right postcentral gyrus	36	−32	56	8.7
Left postcentral gyrus	−28	−30	52	7.1
Right posterior operculum	39	−21	20	6.5
Left posterior operculum	−40	−21	22	4.1
Right cerebellum	32	−80	−38	16.5
Left cerebellum	−34	−80	−38	16.0
Right cerebellum	8	−54	−42	13
Left cerebellum	−6	−56	−44	11.2
**MACM and RS(dmPFC)**				
Ventromedial prefrontal cortex	−4	48	−12	7.5
Dorsomedial prefrontal cortex	2	56	24	8.7
Right amygdala/hippocampus	20	−4	−18	5.3
Left amygdala/hippocampus	−20	−6	−18	6.5
Right inferior frontal gyrus	44	26	−12	4.1
Left inferior frontal gyrus	−48	28	−6	8
Left anterior insula	−36	20	−24	4.5
Posterior cingulate cortex	−4	−36	40	3.3
Retrosplenial cortex	−6	−56	8	5.1
Right temporo-parietal junction	54	−70	20	6.4
Left temporo-parietal junction	−46	−74	36	6.9
Left middle temporal gyrus	−62	−36	2	5.2
Right temporal pole	36	18	−20	3.6
Left temporal pole	−36	20	−24	4.5

Table shows coordinates derived from respective cluster peaks (x, y, z) and Z-scores (Z).

### Functional connectivity: difference analyses between seeds

**Figure 3:**
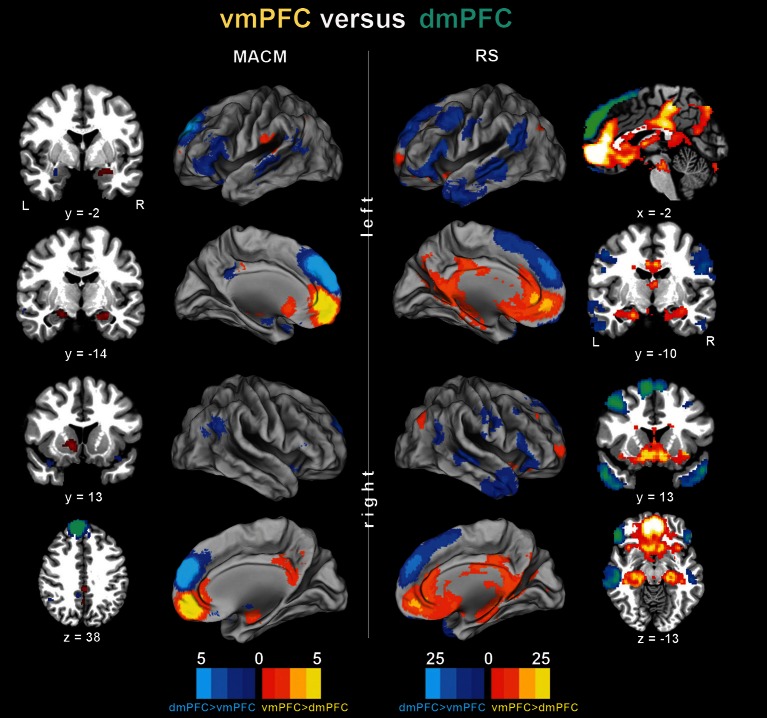
**Functional connectivity differences between the vmPFC and dmPFC seeds.** Connectivity differences between the seeds individually determined using meta-analytic connectivity modeling (MACM) and resting-state (RS) analyses. The color bars on the bottom represent *Z*-values. All images were rendered using Caret. Coordinates in MNI space.

To subsequently determine which brain areas are more strongly coupled with one seed than the other seed, we computed MACM and RS connectivity differences between both seeds (Figure 3). In MACM analyses, the brain areas more strongly coupled with the vmPFC than dmPFC comprised the bilateral vmPFC extending into the ACC, HC (extending into the AM on the right), PCC, and RSC, as well as the left NAc and pOP. In RS analyses, the brain areas more strongly coupled with the vmPFC than dmPFC comprised the bilateral vmPFC, HC, ACC, pMCC, PCC, RSC, Prec, NAc, AI, midbrain/pons, thalamus, visual cortex, posterior lateral parietal cortex, and Cer (not depicted). In MACM analyses, the brain areas more strongly coupled with the dmPFC than vmPFC, in turn, comprised the bilateral PCC, IFG, TPJ, and TP, as well as the left AM and MTG. In RS analyses, the brain areas more strongly coupled with the dmPFC than vmPFC comprised the bilateral orbitofrontal cortex, IFG, MTG, TPJ, TP, PreG, PoG, and Cer (not depicted).

### Functional connectivity: cross-validation by conjunction analyses

The main goal of our study was the functional connectivity of each seed that is consistent across both types of connectivity analysis (i.e., MACM and RS). Convergence of both approaches should reveal connectivity that is consistently observed across two different states of brain function, that is, during specific task performance (MACM) and in the absence of an externally structured task (RS). To thus test for brain areas congruently connected to either seed across both types of connectivity, we computed the conjunction across the respective MACM and RS analyses (Figure 2 and Tables 1, 2). These conjunction analyses of each seed revealed the same set of brain areas as the respective MACM analysis, except for absent vmPFC connectivity to the operculum.

**Figure 4:**
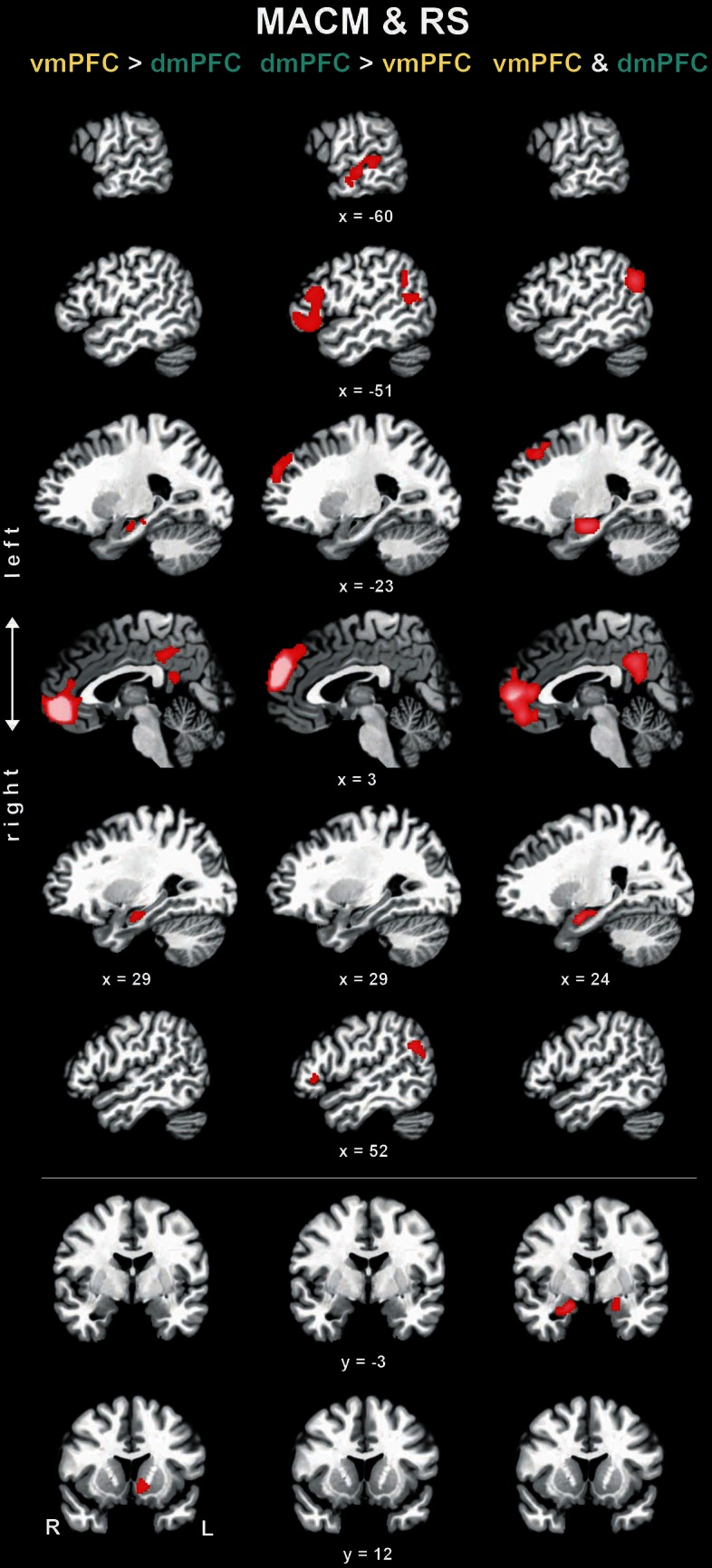
**Difference and conjunction analyses based on congruent functional connectivity of the vmPFC and dmPFC seeds.** Depicts sagittal and coronal brain slices of areas consistently more strongly coupled (left and middle column) with either seed or congruently coupled with both seeds (right column) across meta-analytic connectivity modeling (MACM) and resting-state (RS) analyses. Please refer to Table 3 for activation coordinates. All slices were created using mango (multi-image analysis GUI; http://ric.uthscsa.edu/mango/) on a T1-weighted MNI single subject template. Coordinates in MNI space. </>, difference analysis; &, conjunction analysis; R, right; L, left.

To test for brain areas more strongly coupled with either seed across MACM and RS analyses, we computed the conjunction across the respective MACM- and RS-based difference analyses (Figure 4, Table 3). Across MACM and RS, brain areas congruently more strongly coupled with the vmPFC than dmPFC comprised the bilateral vmPFC extending into the ACC, HC, PCC, and RSC, as well as the left NAc. Across MACM and RS, brain areas congruently more strongly coupled with the dmPFC than vmPFC comprised the bilateral dmPFC, IFG, and TPJ, as well as the left MTG.

**Table 3:** **Difference and conjunction analyses between functional connectivity of the vmPFC and dmPFC seeds**.

**Macroanatomical location**	***x***	***y***	***z***	***Z***
**MACM & RS (vmPFC > dmPFC)**				
Ventromedial prefrontal cortex	2	44	−18	8.1
Right hippocampus	30	−10	−22	3.0
Left hippocampus	−20	−14	−18	2.7
Left nucleus accumbens	−8	18	−4	4.6
Posterior cingulate cortex	4	−38	38	3.2
Retrosplenial cortex	2	−46	18	2.3
Retrosplenial cortex	−12	−58	16	2.9
**MACM & RS (vmPFC < dmPFC)**				
Dorsomedial prefrontal cortex	2	58	12	8.1
Right inferior frontal gyrus	52	28	0	2.1
Left inferior frontal gyrus	−42	40	−10	3.4
Left inferior frontal gyrus	−50	28	18	3.3
Right temporo-parietal junction	56	−54	26	3.8
Left temporo-parietal junction	−50	−52	30	3.0
Left temporo-parietal junction	−50	−56	10	2.5
Left middle temporal gyrus	−60	−22	−8	3.7
**MACM & RS (vmPFC & dmPFC)**				
Ventromedial prefrontal cortex	−4	48	−12	7.5
Frontal pole	−4	56	2	8.4
Left dorsomedial prefrontal cortex	−18	38	46	4.5
Right amygdala/hippocampus	20	−4	−18	5.3
Left amygdala/hippocampus	−24	−12	−20	5.8
Posterior cingulate cortex/retrosplenial cortex	−2	−52	30	6.7
Left temporo-parietal junction	−48	−66	28	6.0

Table shows coordinates derived from respective cluster peaks (x, y, z) and Z-scores (Z). < and > denote difference analyses, while & denotes conjunction analysis.

Finally, the brain areas congruently coupled with the vmPFC and dmPFC across both MACM and RS analyses comprised the bilateral vmPFC, frontal pole, AM/HC, and PCC/RSC, as well as the left dmPFC and TPJ.

### Functional profiling of the seeds

**Figure 5:**
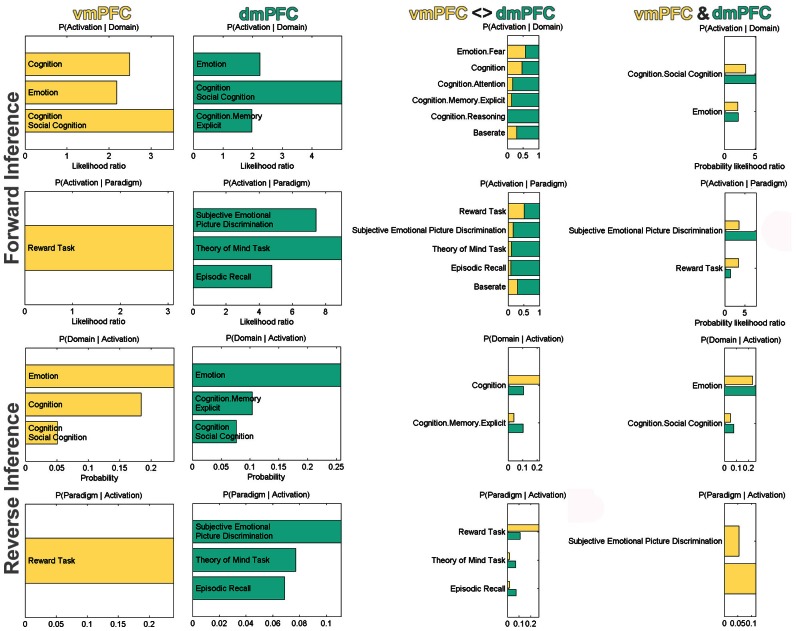
**Functional profiling of the vmPFC and dmPFC seeds.** Significant associations with psychological terms (behavioral domains and paradigm classes) from BrainMap meta-data. Functional profiling was performed as individual, difference, and conjunction analysis. Forward inference determines above-chance brain activity given the presence of a psychological term, while reverse inference determines the above-chance probability of a psychological term given observed brain activity. The base rate denotes the general probability of finding BrainMap activation in the seed. The *x*-axis indicates relative probability values.

After the characterization using connectivity analyses, we also conducted a functional characterization of the vmPFC and dmPFC seeds by determining their significant associations with BrainMap taxonomic categories (Figure 5). For robustness, we focused on taxonomic associations that are significant in both the forward and reverse inference analysis. Forward inference derives brain activity from a psychological term, whereas reverse inference derives a psychological term from brain activity (see Methods section). Accordingly, activity increases in the vmPFC were consistently associated with tasks related to general cognition, social cognition, as well as emotion and reward processing. Note that BrainMap experiments are labeled as related to general cognition mostly if they do not fit into any of the more specific categories. Activity increases in the dmPFC were consistently associated with tasks related to social cognition, theory of mind (i.e., perspective-taking), episodic memory retrieval, as well as processing emotion, also when derived from faces. Note that BrainMap experiments labeled as related to “Episodic Recall” are very likely to be also labeled as “Cognition.Memory.Explicit” rendering these two taxonomic subcategories highly inter-related. When quantifying the taxonomic associations of the seeds relative to each other, the vmPFC (versus dmPFC) was more consistently associated with reward processing and general cognition, while the dmPFC (versus vmPFC) was more consistently associated with (episodic) memory retrieval and theory-of-mind processing. Finally, the taxonomic associations consistent across both vmPFC and dmPFC comprised tasks related to social, emotional, and facial (i.e., “Subjective Emotional Picture Discrimination”) processing.

## Discussion

We examined the widely assumed but not directly tested ventrodorsal differentiation of the mPFC in social cognition. This test of segregation was based on a ventral and dorsal mPFC region that are both consistently related to perspective-taking as a prototypical instance of social cognition. The seeds were analyzed using two ways of functional connectivity analyses by independently delineating task-related meta-analytic connectivity modeling (MACM, Eickhoff et al., 2011) and task-unrelated resting-state correlations (RS, Biswal et al., 1995). Additionally, it was tested whether the seeds were differentially associated with psychological terms from BrainMap meta-data using forward and reverse inference. In both MACM and RS analyses, the vmPFC was more strongly connected with the nucleus accumbens (NAc), hippocampus (HC), posterior cingulate cortex (PCC), and retrosplenial cortex (RSC), while the dmPFC was more strongly connected with the inferior frontal gyrus (IFG), temporo-parietal junction (TPJ), and middle temporal gyrus (MTG). In both functional decoding analyses, the vmPFC was selectively associated with reward related tasks, while the dmPFC was selectively associated with perspective-taking and episodic memory retrieval tasks. Importantly, both vmPFC and dmPFC were functionally associated with social, emotional, and facial processing. In sum, the vmPFC was thus more closely connected to limbic and reward-related medial brain areas as well as functionally associated with processing approach- and avoidance-relevant stimuli. In contrast, the dmPFC was more connected to higher associative cortical areas as well as functionally associated with processing mental states and episodic memory.

### Connectional evidence for the segregation between the vmPFC and dmPFC

Our convergent connectivity results across MACM and RS analyses derived from the vmPFC and dmPFC seeds agree well with many earlier findings in humans and monkeys. Importantly, the vmPFC and dmPFC have been found to be extensively inter-connected in axonal tracing studies in monkeys (Barbas et al., 1999; Saleem et al., 2008), consistent with our results. In the following, we will compare the present connectivity differences between the vmPFC and dmPFC with earlier findings using other connectivity measures in humans and monkeys.

The vmPFC, on the one hand, was more strongly connected to the NAc, HC, PCC, and RSC across two different types of functional connectivity analysis in the present study. Indeed, the vmPFC, but not dmPFC, has been observed to have monosynaptical connections with the ventral striatum (VS, which anatomically includes the NAc) in axonal tracing studies in monkeys (Haber et al., 1995; Ferry et al., 2000). This is consistent with our results and probabilistic diffusion tensor imaging (DTI) tractography in humans and monkeys (Croxson et al., 2005) that quantified the VS to be substantially more likely connected to the vmPFC than dmPFC in both species. This DTI study further estimated the vmPFC to be only slightly more connected to the amygdala (AM) than the dmPFC in monkeys and humans (cf. Bzdok et al., 2012a), in line with the present AM connectivity to both vmPFC and dmPFC. Importantly, roughly balanced connectivity to the AM challenges the frequently proposed vmPFC-dmPFC distinction as emotional versus cognitive. Although monkey tracing studies indicated that the entire medial wall of the prefrontal cortex has amygdalar and cingulate connections, the most ventral part of the mPFC received strongest connections from most limbic areas, including the HC (Carmichael and Price, 1995). This concurs with our results and a RS connectivity analysis of the human HC showing more correlation with the vmPFC than dmPFC (Vincent et al., 2006). Additionally, fibers from the medial temporal lobe (including the AM and HC) entered the mostly ventral medial partial cortex, including the RSC, as observed using DTI tractography in humans (Greicius et al., 2009). Our results are in line with monkey tracing studies showing that mostly the vmPFC but also dmPFC are directly connected to the PCC (Carmichael and Price, 1995) and RSC (Vann et al., 2009). Conversely, the PCC and RSC (but not the more dorsocaudal precuneus) were mostly connected to limbic regions and the vmPFC in a comparative RS study in monkeys and humans (Margulies et al., 2009). Concluding from previous and present connectivity findings, *the vmPFC is preferentially connected with limbic and reward-related medial brain areas.*

The dmPFC, on the other hand, was more strongly connected to the TPJ, MTG, and IFG across two different types of functional connectivity analysis in the present study. Using DTI tractography in humans the vmPFC and dmPFC have been observed to be connected to the TPJ, which in turn was connected to the MTG (Caspers et al., 2011). Although we also found convergent functional connectivity of the vmPFC and especially dmPFC to the TPJ, monosynaptical connections from the anterior prefrontal cortex to the TPJ *might* be absent in monkeys (for discussion, see Caspers et al., 2011). Existence of mPFC-TPJ connectivity in humans is supported by the present results, while our methodological approach cannot distinguish mono- and polysynaptical connections. Our results therefore cannot contribute to the more general question whether direct mPFC-TPJ connections exist in humans but not monkeys. The TPJ and IFG, both relatively more connected to the dmPFC in our study, were also reported to be connected in an axonal tracing study in monkeys (Petrides and Pandya, 2009) and in a DTI study in humans (Frey et al., 2008). Both the vmPFC and dmPFC are further known to have direct connections with the IFG and MTG based on monkey tracing data (Yeterian et al., 2012). In contrast, those two target areas were more strongly connected to the dmPFC in our functional connectivity analyses. Thus, axonal connections between the vmPFC and the IFG and MTG presumably existing in humans might be less important for social-cognitive processing than those of the dmPFC. Similarly, although DTI tractography in humans (Greicius et al., 2009) and axonal tracing in monkeys (Cavada and Goldman-Rakic, 1989) have identified fiber bundles connecting the dmPFC with the more dorsal and posterior medial parietal cortex (precuneus), this was not reflected by our functional connectivity results. Concluding from previous and present connectivity findings, *the dmPFC is preferentially connected with high association and heteromodal cortical areas of the lateral frontal, temporal, and parietal lobe.* More globally, most of the present *functional connectivity* findings of the human vmPFC and dmPFC concur very well with knowledge describing *structural connectivity* in the monkey and human brain. However, our results also show that known axonal connections between the mPFC and other parts of the brain are not always reflected in functional connectivity analyses.

### Integrative segregation between the vmPFC and dmPFC

After discussing the connectivity differences between the vmPFC and dmPFC, we will now discuss the previously proposed functional properties of their respective connectivity targets (cf. Fuster, 2001). The vmPFC was more connected to the NAc, HC, PCC, and RSC. The NAc is thought to be linked to reward mechanisms that may not only modulate motivated behavior towards basic survival needs, such as food and sex, but also towards salient social cues (cf. Kampe et al., 2001; Cardinal et al., 2002; Walter et al., 2005; Schilbach et al., 2010). Neuroimaging research indeed ascribed complex reward functions to the NAc, such as the evaluation of reward expectancy in social, monetary, or drug rewards (Schultz et al., 1997; Kampe et al., 2001; Rademacher et al., 2010; Bzdok et al., 2011). The HC, in turn, is well known to be involved in memory and spatial navigation in animals and humans (von Bechterew, 1900; Scoville and Milner, 1957; O'Keefe and Dostrovsky, 1971; Maguire et al., 2000). As to the PCC and RSC, electrophysiological research in animals implicated the PCC in strategic selection (Pearson et al., 2009), risk assessment (McCoy and Platt, 2005), and outcome-contingent behavioral modulation (Hayden et al., 2008), while the RSC was implicated in navigation and approach-avoidance behavior (Vann et al., 2009). Considering only the previously reported functional properties of the here more strongly connected nodes, the vmPFC can be assumed to integrate a subnetwork (i.e., the brain areas relatively more connect to the vmPFC, excluding the vmPFC seed itself) modulating online approach-avoidance behavior by memory-informed reward and risk estimation of self-relevant environmental stimuli.

In contrast, the dmPFC was more connected to the IFG, TPJ, and MTG. As these subnetwork nodes (i.e., the brain areas relatively more connected to the dmPFC, excluding the dmPFC seed itself) are highly associative and heteromodal, there is less clarity and agreement about their discrete functional contributions. As a side note, the mere difference in the association level between the vmPFC's and dmPFC's subnetworks already indicates functional segregation (Mesulam, 1998). Moreover, the entire set of dmPFC-linked regions is well known to concomitantly increase and decrease metabolic activity as a cohesive unit, as lateral components of the so-called “default mode network” (Gusnard et al., 2001; Laird et al., 2009b; Spreng et al., 2009; Mar, 2011; Bzdok et al., 2012c; Schilbach et al., 2012). In fact, it is interesting to note that the vmPFC is more strongly connected to medial components of the default mode network (i.e., HC, PCC, RSC), whereas the dmPFC is more strongly connected to its lateral components (i.e., IFG, TPJ, and MTG). This dmPFC subnetwork was repeatedly related to self-focused reflection (Andrews-Hanna et al., 2010), contemplation of others' (Mar, 2011) and one's own (Lombardo et al., 2009) mental states, mental navigation of the body in space (Maguire et al., 1997), semantic processing (Binder et al., 2009), as well as scene construction processes when envisioning past, fictitious, and future events (Hassabis et al., 2007; Spreng et al., 2009; Bzdok et al., 2013). Interestingly, the neuroimaging studies related to processing semantic information (Binder et al., 2009), autobiographical (Spreng et al., 2009) and fictitious (Hassabis et al., 2007) events observed neural activity increases in both the vmPFC and dmPFC, although the respective neural networks resemble much more the dmPFC (rather than vmPFC) subnetwork. The conjunction of previous and present findings suggests that the dmPFC integrates a network involved in self- or other-related, largely sensory-independent, highly abstract (hence, less tangible) processes across time, space, and content domains. Importantly, the previously proposed vmPFC-dmPFC distinction as outcome-oriented versus goal-oriented is challenged by our results that support outcome-oriented vmPFC processing but not specifically goal-oriented dmPFC processing. It is also important to note that both the vmPFC and dmPFC are closely related to memory retrieval as indicated by converging functional connectivity (across MACM and RS) to the HC. However, the memory-retrieved information appears to be bound with less complex neural processes in the vmPFC versus dmPFC as indicated by functional association with, for instance, less complex reward processes versus more complex perspective-taking processes.

Additionally, the here identified subnetworks belonging to the vmPFC and dmPFC corroborate an earlier hierarchical clustering analysis based on an fMRI study (Andrews-Hanna et al., 2010). In particular, seed regions were derived from comparing future versus present self-related thinking in bidirectional fMRI contrasts. Subsequent resting-state analyses of these seed regions allowed clustering into a vmPFC-associated subnetwork, including the HC and PCC/RSC, and a dmPFC-associated subnetwork, including the TPJ and MTG. The fMRI data then related, respectively the vmPFC and dmPFC subnetworks to thinking about present and future self, in line with our functional decoding results. Put differently, the vmPFC might be more closely associated with orchestrating adapted behavior by bottom-up-driven processing of “what matters now”, which might be top-down modulated by more dmPFC subserved higher reflective and hypothetical processing.

### Morphological evidence for the segregation between the vmPFC and dmPFC

It may be instructive to acknowledge the relationship between the present findings on social cognition in mPFC subregions and the recently increasing evidence for the “social brain” that might have coevolved with the complexity of social relationships (Jolly, 1966; Humphrey, 1978; Byrne and Whiten, 1988; Dunbar, 1998; Dunbar and Shultz, 2007). Most importantly, independent whole-brain analyses from *structural* neuroimaging studies related the gray-matter volume (GMV) of the vmPFC to indices of social competence and social network complexity in both humans and monkeys (Lebreton et al., 2009; Powell et al., 2010; Lewis et al., 2011; Sallet et al., 2011). To our knowledge, none of these four correlations have been found yet for the dmPFC. Consequently, vmPFC, rather than dmPFC, anatomy appears to predict an individual's social behavioral dispositions and social network properties, although we found both regions to be congruently associated with social, emotional, and facial processing.

Such brain-behavior correlations in humans were also shown for the brain areas preferentially connected to the vmPFC or dmPFC in the present analysis. As to the vmPFC subnetwork, the GMV of the vmPFC and VS correlated with indices of social reward attitudes and behavior (Lebreton et al., 2009), concurring with vmPFC's relation to the NAc and reward-related tasks. Additionally, the GMV of the entorhinal cortex (connectionally and functionally closely coupled with the HC) correlated with social network size (Kanai et al., 2012), concurring with vmPFC's connectivity to the HC. Further, vmPFC and PCC/Prec GMV correlated with social network size (Lewis et al., 2011), concurring with vmPFC's stronger connectivity to the PCC. As to the dmPFC subnetwork, the GMV of the TPJ and MTG correlated with social network size (Kanai et al., 2012), while the GMV of the TPJ and IFG correlated with perspective-taking competence (Lewis et al., 2011). Moreover, the GMV of the amygdala, connected to both vmPFC and dmPFC, correlated negatively with social phobia (Irle et al., 2010) and positively with social network size (Bickart et al., 2010).

The conjunction of these recent brain-behavior correlations and the present results allow several conclusions. With respect to our seeds, inter-individual differences in social skills or social networks were most often related to morphological differences in the human and monkey vmPFC, in stark contrast to the dmPFC. With respect to the seeds' subnetworks, the reported brain-behavior correlations were roughly equally related to the more vmPFC or dmPFC connected brain areas. With respect to the type of social variable, morphological differences related to either social skills or networks do not seem to be preferentially associated with the more vmPFC or dmPFC connected brain areas.

The conclusions prompt the hypothesis that the dmPFC subserves a domain-independent neural process important for, but not specific to, social cognition. Indeed, the present results support the dmPFC's possible involvement in domain-overarching computational mechanisms given its connections to highly associative brain areas and functionally relation to different complex psychological processes. Although vmPFC and dmPFC were associated with social, emotional, and facial processing, the dmPFC probably processes these types of information on a higher level of abstraction.

### Neuropsychological evidence for the segregation between the vmPFC and dmPFC

The conclusions derived from our findings are corroborated by brain lesion data. Consistent with the functional association of the vmPFC with reward processing as well as with a role in predominantly *self-related* behavior guided by stimulus evaluation and reward-learning, a voxel-based lesion-symptom mapping (VLSM) study in 344 neurological patients demonstrated functional-anatomical specificity of the vmPFC for value-based decision-making (Gläscher et al., 2012). However, vmPFC damage in humans also impairs an array of predominantly *other-related* socio-emotional processes. More specifically, consistent with vmPFC's connectivity to both the limbic system and the dmPFC, vmPFC lesions appear to impair the *integration* of (other-related) higher social, basic emotional, and facial processes, rather than any of these three classes of neural processes per se (Bzdok et al., 2012b). This is indicated by (1) disrupted emotion recognition from faces (Hornak et al., 1996) despite intact face recognition (Shamay-Tsoory et al., 2005; Monte et al., 2012), (2) sociopathic behavior in every-day life (Blair and Cipolotti, 2000) despite intact abstract reflection of social phenomena (Saver and Damasio, 1991; Damasio, 1996; Young et al., 2010), (3) disrupted affective but not cognitive perspective-taking (Stone et al., 1998; Stuss et al., 2001; Shamay-Tsoory et al., 2006; Shamay-Tsoory and Aharon-Peretz, 2007), (4) disrupted perspective-taking-based empathy despite intact simpler affective empathy (Shamay-Tsoory et al., 2009), and (5) reduced emotional impact on moral judgments (Koenigs et al., 2007; Young et al., 2010).

Put differently, vmPFC lesion might alter the subset of abstract social processes that require vmPFC-mediated relay of emotional limbic information to the dmPFC, consistent with our connectional and functional results. Indeed, faux detection (i.e., abstract social processing involving emotion processing) is impaired after damage to either the amygdalae (Stone et al., 2003) or the vmPFC (Gregory et al., 2002). The conjunction of previous lesion reports and present results therefore suggests that the vmPFC interweaves more emotional processes (mainly subserved by the limbic system) and more *ambiguous* social thought (probably subserved by the dmPFC) to shape self- and other-related behavioral responses to sensory events in social cognition (Shamay-Tsoory and Aharon-Peretz, 2007; Bzdok et al., 2012b).

Juxtaposing the effects of vmPFC and dmPFC lesions in humans is impeded by the scarcity of circumscribed dmPFC lesions (cf. Mochizuki and Saito, 1990; Duffy and Campbell, 1994; Wilson et al., 2010). Although quite heterogeneous, the few available dmPFC-linked lesion findings consolidate the here derived segregation within the mPFC as a function of reliance on bottom-up versus top-down processing pathways. First, the dmPFC subnetwork was normally recruited in congenitally blind individuals engaged in perspective-taking (Bedny et al., 2009). Therefore, complete lack of visual input does not appear to alter functioning of this high-level area, contrarily to low-level visual cortices. Second, a VLSM study on disturbed sleep (i.e., a state of mind independent of sensory stimulation but dependent on internally generated information) *exclusively* identified the dmPFC (Koenigs et al., 2010). Third, another VLSM study exclusively related the IFG and TPJ, both more strongly connected to the dmPFC in our study, to inner speech (Geva et al., 2011). Taken together, in individuals with an intact central nervous system, the vmPFC versus dmPFC are probably involved in predominantly bottom-up versus top-down mediated processing of social information.

### Neuroimaging evidence for the segregation between the vmPFC and dmPFC

Following the observed functional associations with fear and reward, the vmPFC is likely to process not only external but also visceral stimuli. Indeed, measurements of task-induced brain activity changes in humans confirm our functional decoding results by relating the vmPFC to monitoring others' (Lotze et al., 2007) and one's own (Lane et al., 1997; Phan et al., 2004) emotional responses, that is, other's (external) emotional reactions and one's own (visceral) arousal. Such real or imagined bodily states, believed to be represented in the vmPFC, probably operate as a bioregulatory disposition governing cognition and decision making (Damasio, 1996; Nauta, 1971), in line with the vmPFC's functional association with general cognition and reward processing. An fMRI study, for instance, reported specific vmPFC activity increases during other-initiated joint attention, suggesting representation of the motivational significance of social cues (Schilbach et al., 2010). Consistent with our line of interpretation, vmPFC versus dmPFC activity was moreover shown to reflect actually choice-relevant versus modeled, choice-irrelevant value in a computational fMRI study (Nicolle et al., 2012). The conjunction of previous functional neuroimaging findings and our functional profiling data consolidate the vmPFC's role in processing self- and other-related visceroaffective and motivational information as a guide in ongoing social behavior.

Moreover, the vmPFC and dmPFC were both significantly associated with social, emotional, and facial processing in the present study. This indicates that the vmPFC and dmPFC are not functionally dissociable by selective involvement in social, emotional, or facial processing, although this is frequently proposed. However, the dmPFC, but not vmPFC, was congruently associated with more complex social-cognitive tasks across forward and reverse functional decoding, including perspective-taking and episodic memory retrieval. While the former imposes an other-focused mind set, the latter inherently entails a self-focused mind set (obviously, one can only recall scenes from one's own personal experience). Quantitative functional profiling of the dmPFC therefore indicates that the dmPFC is involved in both self- and other-oriented processing, analogous to the vmPFC. Importantly, the frequently proposed vmPFC-dmPFC distinction as self versus other is challenged by our conclusions.

In particular, consistent with present functional decoding, neural activity in the dmPFC, rather than vmPFC, has been consistently interpreted to underlie inference, representation, and assessment of one's own and others' mental states in functional neuroimaging research (Gusnard et al., 2001; Gallagher and Frith, 2003; Amodio and Frith, 2006; Gilbert et al., 2006; Ochsner, 2008; Van Overwalle, 2009; Bzdok et al., 2012b; Moran et al., 2012). For instance, dmPFC (but not vmPFC) activity was related to the proficiency decline of mental state inference in elderly (Moran et al., 2012), *cognitive regulation* of one's own emotional states (Ochsner et al., 2004b) and *inference* of another person's emotional states (Ochsner et al., 2004a), as well as self-reported (Wagner et al., 2011) and experimentally measured (Zaki et al., 2009) proficiency in emotional state inference. Notably, such self- and other-related conceptualizations cannot be made based on sensory information or general knowledge about the physical world (cf. Premack and Woodruff, 1978; Leslie, 1987; Carruthers, 2009). Thus, mental state inference necessarily relies on the generation of probabilistic internal information. Supported by dmPFC's functional association with episodic memory retrieval, such prima vista non-mnemonic construction processes are likely to be subserved by the neural network underlying retrieval of past and imagination of future scenes as indicated by recent neuroimaging experiments and meta-analyses (Schacter et al., 2007; Spreng et al., 2009; Andrews-Hanna et al., 2010; Rabin et al., 2010; Bzdok et al., 2012c). Constructing such probabilistic scenes is further believed to necessarily drawn on semantic knowledge retrieval (Binder et al., 1999; Bar, 2007; Suddendorf and Corballis, 2007; Carruthers, 2009; Bzdok et al., 2012c). This would be in line with left lateralization of the dmPFC subnetwork typical of semantic processing (Binder et al., 2009). The conjunction of previous functional neuroimaging findings and present neuroinformatic findings congruently characterizes the dmPFC as a “mental sketchpad” (Goldman-Rakic, 1996) potentially implicated in modeling and binding plausible self- and other-related scenarios instructed by semantic concepts in social cognition. Again, such sensory-independent *de novo* generation of meaning representations can only be expected from highly associative, integrative brain areas such as those of the dmPFC subnetwork (Mesulam, 1998), as opposed to the vmPFC subnetwork.

## Conclusion

Although the human mPFC is neither uniquely nor solely devoted to social cognition, its central role in navigating the interpersonal space is probably one of the most often replicated findings in functional neuroimaging research. However, the strength of cognitive neuroscience comes from investigating an identical phenomenon from various conceptual and methodological perspectives (cf. Feyerabend, 1975). We therefore re-examined the widely assumed ventrodorsal functional segregation of the mPFC in social cognition in a bottom-up approach and integrated the ensuing results with different literatures. As a result of this, we comprehensively characterized *both* the vmPFC and dmPFC as relevant for self- and other-focused as well as social, emotional, and facial processing. More specifically, the vmPFC subserves predominantly non-ambiguous subjective-value-related evaluative processes driven by bottom-up pathways, whereas the dmPFC subserves predominantly ambiguous amodal metacognitive processes driven by top-down pathways. These conclusions amend a number of earlier accounts on the division of labor between ventral and dorsal aspects of the mPFC in social cognition. Ultimately, the integration of external stimulation and internal generation driven processes in the mPFC is a part of what determines social behavior.

### Conflict of interest statement

The authors declare that the research was conducted in the absence of any commercial or financial relationships that could be construed as a potential conflict of interest.
